# Analysis of the *TSC1*and *TSC2*genes in sporadic renal cell carcinomas

**DOI:** 10.1054/bjoc.2001.2072

**Published:** 2001-10

**Authors:** L Parry, J H Maynard, A Patel, S C Clifford, C Morrissey, E R Maher, J P Cheadle, J R Sampson

**Affiliations:** Institute of Medical Genetics, University of Wales College of Medicine, Cardiff, CF14 4XN, UK; Section of Medical and Molecular Genetics, Department of Paediatrics and Child Health, University of Birmingham, Birmingham, UK

**Keywords:** *TSC1*, *TSC2*, sporadic renal cell carcinoma

## Abstract

The genetic events involved in the aetiology of non-clear-cell renal cell carcinoma (RCC) and a proportion of clear cell RCC remain to be defined. Germline mutations of the *TSC1*and *TSC2*genes cause tuberous sclerosis (TSC), a multi-system hamartoma syndrome that is also associated with RCC. We assessed 17 sporadic clear cell RCCs with a previously identified *VHL*mutation, 15 clear-cell RCCs without an identified *VHL*mutation and 15 non-clear-cell RCCs for loss of heterozygosity (LOH) at chromosomes 9q34 and 16p13.3, the chromosomal locations of *TSC1*and *TSC2*. LOH was detected in 4/9, 1/11 and 3/13 cases informative at both loci. SSCP analysis of the whole coding region of the retained allele did not reveal any cases with a detectable intragenic second somatic mutation. Furthermore, RT-PCR analysis of *TSC1*and *TSC2*on total RNA from 8 clear-cell RCC cell lines confirmed expression of both TSC genes. These data indicate that biallelic inactivation of *TSC1*or *TSC2*is not frequent in sporadic RCC and suggests that the molecular mechanisms of renal carcinogenesis in TSC are likely to be distinct. © 2001 Cancer Research Campaignhttp://www.bjcancer.com  http://www.bjcancer.com


*British Journal of Cancer* (2001) **85**(8), 1226–1230
© 2001 Cancer Research Campaign
doi: 10.1054/ bjoc.2001.2072, available online at http://www.idealibrary.com on IDEAL

http://www.bjcancer.com

### Analysis of the *TSC1* and *TSC2* genes in sporadic renal cell carcinomas

**L Parry[1], JH Maynard[1], A Patel[1], SC Clifford[2], C Morrissey[2], ER Maher[2], JP Cheadle[1] and JR Sampson[1]**

[1]Institute of Medical Genetics, University of Wales College of Medicine, Cardiff, CF14 4XN, UK; [2]Section of Medical and Molecular Genetics, Department of Paediatrics and Child Health, University of Birmingham, Birmingham, UK

**Summary** The genetic events involved in the aetiology of non-clear-cell renal cell carcinoma (RCC) and a proportion of clear cell RCC remain to be defined. Germline mutations of the *TSC1* and *TSC2* genes cause tuberous sclerosis (TSC), a multi-system hamartoma syndrome that is also associated with RCC. We assessed 17 sporadic clear cell RCCs with a previously identified *VHL* mutation, 15 clear-cell RCCs without an identified *VHL* mutation and 15 non-clear-cell RCCs for loss of heterozygosity (LOH) at chromosomes 9q34 and 16p13.3, the chromosomal locations of *TSC1* and *TSC2*. LOH was detected in 4/9, 1/11 and 3/13 cases informative at both loci. SSCP analysis of the whole coding region of the retained allele did not reveal any cases with a detectable intragenic second somatic mutation. Furthermore, RT-PCR analysis of *TSC1* and *TSC2* on total RNA from 8 clear-cell RCC cell lines confirmed expression of both TSC genes. These data indicate that biallelic inactivation of *TSC1* or *TSC2* is not frequent in sporadic RCC and suggests that the molecular mechanisms of renal carcinogenesis in TSC are likely to be distinct. © 2001 Cancer Research Campaign http://www.bjcancer.com

**Keywords**: *TSC1; TSC2*; sporadic renal cell carcinoma

The molecular genetic events leading to renal cell carcinoma (RCC) are not fully understood. Recurrent regions of deletion on chromosomes 3p, 4q, 6q, 8p, 9p and amplifications on 17q and Xq have been revealed by comparative genomic hybridisation (CGH) (Verdorfer et al, 1998; Bissig et al, 1999) and loss of heterozygosity (LOH) studies (Thrash-Bingham et al, 1995), as have alterations on 2, 3, 9–12, 16, 17 and 18 by restriction landmark genomic scanning (RLGS) (Cho et al, 1998a,b). The molecular pathology of RCC varies between histopathological subtypes. Thus chromosome 3p allele loss is the most frequent alteration in clear cell RCC (which accounts for ~80% of tumours) but is rare in non-clear-cell RCC. Several known and putative tumour suppressor genes (TSGs) map to 3p and both the von Hippel–Lindau (*VHL*) (Latif et al, 1993) TSG and further gene(s) at 3p12–p21 have been implicated in clear cell RCC. *VHL* is mutated, deleted or hypermethylated in up to 70% of sporadic clear cell RCCs in addition to von Hippel–Lindau disease associated renal cell carcinoma (Prowse et al, 1997), but does not appear to play a significant role in papillary or other non-clear-cell cancers (Foster et al, 1994; Gnarra et al, 1994; Herman et al, 1994; Shuin et al, 1994; Clifford et al, 1998). Inactivation of 3p12–p21 TSG(s) has been implicated in most clear-cell RCC irrespective of VHL status, and to date no differences in molecular pathology have been identified between clear-cell RCC with and without VHL inactivation (Clifford et al, 1998, 1999).

A role for the *cMET* gene that encodes the receptor for hepatocyte growth factor has been demonstrated in type 1 papillary RCC, since constitutionally activating germline missense mutations occur in a rare hereditary form of papillary RCC as do somatic mutations in some sporadic cancers (Schmidt et al, 1997). However the genes involved in the majority of non-clear-cell RCC remain to be defined. *TSC1* (van Slegtenhorst et al, 1997) and *TSC2* (European Chromosome 16 Tuberous Sclerosis Consortium, 1993) are Knudson-type TSGs that are constitutionally mutated in the hereditary disorder tuberous sclerosis (TSC). Renal angiomyolipomas are found in most affected individuals, but there also appears to be a less frequent association with RCC and several reports have described multifocal and bilateral disease in unusually young patients, suggesting a possible role for the TSC genes in RCC (Sampson et al, 1995; Bjornsson et al, 1996; Al-Saleem et al, 1998). The identification of LOH or intragenic mutation affecting the wild-type allele of *TSC1* or *TSC2* in TSC-associated RCC has further supported this hypothesis (Bjornsson et al, 1996; van Slegtenhorst et al, 1997; Al-Saleem et al, 1998). In rodent models there is already direct evidence for a role of the *TSC1* and *TSC2* orthologues in RCC. The Eker rat develops multifocal renal cystadenoma and carcinoma and carries a truncating germline mutation of the *Tsc2* gene (Kobayashi et al, 1995). Heterozygous engineered *Tsc1* and *Tsc2* knockout mice develop a similar renal cystadenoma/carcinoma phenotype (Kobayashi et al, 1999; Onda et al, 1999; Kwiatowski DJ, personal communication). Tumours from the mutant animals show somatic second hit mutations of the corresponding *Tsc1* or *Tsc2* wild-type allele (Yeung et al, 1995; Kobayashi et al, 1997, 1999; Onda et al, 1999). Bi-allelic somatic mutations of *Tsc1* and *Tsc2* have also been reported in chemically induced RCCs in non-Eker rats (Urakami et al, 1997; Satake et al, 1999). However, a comprehensive study of *TSC1* and *TSC2* in sporadic human RCC has not yet been reported. We therefore undertook a systematic molecular genetic study of the *TSC1* and *TSC2* genes in different types of sporadic human RCC.

*Received 4 December 2000*
*Revised 19 July 2001*
*Accepted 24 July 2001*

*Correspondence to:* JR Sampson

### MATERIALS AND METHODS

### Tumour and constitutional DNA samples

47 paired sporadic renal cell carcinomas and constitutional DNA samples were studied. These comprised 17 clear-cell RCCs known to harbour *VHL* mutations, 15 clear-cell RCCs in which no *VHL* mutation had been detected (Foster et al, 1994; Clifford et al, 1998) and 15 non-clear-cell RCCs. All patient samples were obtained with consent for molecular genetic analysis.

### RNA from clear cell-RCC cell lines

Total RNA was extracted from 8 clear-cell RCC cell lines, CAKI1, KTCL26, SKRC18, SKRC39, SKRC45, SKRC47, SKRC52 and SKRC54 using the Qiagen RNeasy RNA extraction kit.

### LOH analysis

7 polymorphisms at the *TSC1* locus and 7 at the *TSC2* locus that we have described previously (Parry et al, 2000) were genotyped to assay for LOH in paired tumour and constitutional DNA samples. The *TSC1* markers PM4 and PM2 are situated 50 kb and 5 kb telomeric to the gene respectively, PM1 is located in exon 9, markers 'exon 14' and 'exon 22' are RFLPs, the intron 21 polymorphism is a mononucleotide repeat and PM5 is 50 kb centromeric to the gene. The *TSC2* marker LP1 is 95 kb telomeric to *TSC2*, IVS8 is in intron 8, LP10 is in intron 10, exon 40 contains an RFLP, Kg8 lies within the 3′ UTR of *PKD1* with EJ1 and LP7 1.5 kb and 150 kb centromeric, respectively. PCR amplification of tumour and constitutional DNA samples was carried out in parallel in 96-well microtitre plates (Hybaid). Each 50 µl reaction contained 100 ng DNA, 25 pmoles primer (supplied by Oswel DNA Services), 0.2 mM dNTP (Boehringer Mannheim), 5 µl reaction buffer (100 mM Tris pH 8.3, 500 mM KCI, 15 mM $MgCl_2$, 0.01% gelatin (Cetus)) and 1 U AmpliTaq Gold Polymerase (Cetus). Cycling conditions were 94˚C 10 min, followed by 37 cycles of annealing temperature (55–60˚C) 1 min, 72˚C 1 min, 94˚C 30 s, and a final step of 72˚C 10 min. For autoradiography reverse primers were end labelled with $\gamma^{33}$ dATP (Amersham) using T4 polynucleotide kinase (Life Technologies) according to manufacturers instructions and the products were electrophoresed on 6% polyacrylamide gels (National Diagnostics). The *TSC1* exon 14 1556 A/G polymorphism, the *TSC1* exon 22 3050 C/T polymorphism and the *TSC2* exon 40 1734 T/C polymorphism were assayed by digestion of 10 µl amplified product with the enzymes NIaIV, HaeIII and EcoRV respectively and visualisation on 3% agarose gels stained with ethidium bromide. LOH was determined by visual inspection of alleles from normal and tumour DNA samples by 3 independent observers and samples scored positive by all observers were reamplified and assessed again.

### SSCP analysis and sequencing

In cases exhibiting LOH at the *TSC1* or *TSC2* locus, all known coding exons and exon flanking sequences of the corresponding retained allele were screened by SSCP for evidence of intragenic somatic mutations. Primer sequences and annealing temperatures used are available at the Cardiff-Rotterdam Tuberous Sclerosis Mutation Database Website (www.uwcm.ac.uk/uwcm/mg/tsc_db/pcrpub.html). Amplification reactions were carried out as previously described (Jones et al, 1997). SSCP was performed on

**Table 1** Informativity and LOH of RCCs

| | No. informative and No. showing LOH in parenthesis | | | |
|---|---|---|---|---|
| **Tumour type** | **No.** | ***TSC1*** | ***TSC2*** | ***TSC1* and *TSC2*** |
| Clear cell carcinoma with a *VHL* mutation | 17 | 12 (3) | 14 (1) | 9 (4) |
| Clear cell carcinoma without a *VHL* mutation | 15 | 12 (1) | 14 (0) | 11 (1) |
| Non clear cell carcinoma | 15 | 14 (3) | 14 (0) | 13 (3) |
| Total | 47 | 38 (7) | 42 (1) | 33 (8) |

**Table 2** RCCs showing LOH in the *TSC1* and *TSC2* regions

***TSC1***

| Tumour | Patient | PM4 | PM2 | PM1 | Exon 14 | Intron 21 | Exon 22 | PM5 |
|---|---|---|---|---|---|---|---|---|
| CC-*VHL* mut | 8 | + | NI | NI | NI | NI | NI | + |
| CC-*VHL* mut | 10 | + | + | NI | NI | NI | NI | NI |
| CC-*VHL* mut | 180 | – | – | NI | NI | – | NI | + |
| CC-*VHL* no mut | 6 | NI | + | NI | NI | NI | NI | NI |
| Non-CC | 128 | + | NI | NI | + | NI | NI | + |
| Non-CC | 287 | + | NI | NI | NI | + | NI | NI |
| Non-CC | 297 | + | + | NI | NI | NI | NI | NI |

***TSC2***

| Tumour | Patient | LP1 | IVS8 | LP10 | EXON 40 | EJ1 | Kg8 | LP7 |
|---|---|---|---|---|---|---|---|---|
| CC-*VHL* mut | 239 | – | NI | NI | NI | NI | + | NI |

CC-*VHL* mut – clear cell carcinoma with a mutation in the *VHL* gene; CC-*VHL* no mut – clear cell carcinoma with no identified mutation in the *VHL* gene; Non-CC – non clear cell renal carcinoma; + LOH detected; – No LOH and NI not informative. Shaded boxes indicate intragenic markers. Markers orientated from telomere towards centromere (left to right).

© 2001 Cancer Research Campaign 

4 µl PCR product diluted 1:10 with gel loading buffer (95% formamide, 20 mM EDTA, 0.05% Bromophenol blue, 0.05% Xylene cyanol). Samples were denatured at 94˚C for 2 min and immediately loaded (5 h intervals) on a 0.8 mm MDE gel (Flowgen). Electrophoresis was performed in 0.6% TBE at 20 W for 18 h at room temperature. Products were visualised by standard silver staining (Jones et al, 1997). PCR products of samples displaying variant band patterns were sequenced using either the Sequenase PCR Product Sequencing kit (Amersham) or the Thermosequenase cycle sequencing kit (Amersham).

### Reverse-transcription (RT)-PCR

Synthesis of the first strand cDNA, was performed on 50 ng of total RNA using the Superscript™II kit (Life Technologies). PCR was performed using 1 µl of the first strand cDNA product as a template. To assess expression of *TSC1*, the primers TS1RTX14F (5′-TGGATTCTGCAAGACCATGT-3′) and TS1RTX14R (5′-CTGCTGTGGTGATCTCAGAAA-3′) from exon 14 were used, for *TSC2* the primers TS2RTX14F (5′-TGCTCATCAACAGGC AGTTC-3′) and TS2RTX14R (5′-GCCACATCCCTTTCTTCCA-3′) from exon 14 and TS2RTX41F (5′-CACCGATATCTACCC-CTCCA-3′) and TS2RTX41R (5′-GACAGGCAATACCGTCCA AG-3′) from exon 40 were used.

### RESULTS

38 of the 47 RCC's were informative for at least one marker in the *TSC1* region and seven showed LOH (Tables 1 and 2). 42 tumours were informative for one marker or more in the *TSC2* region and

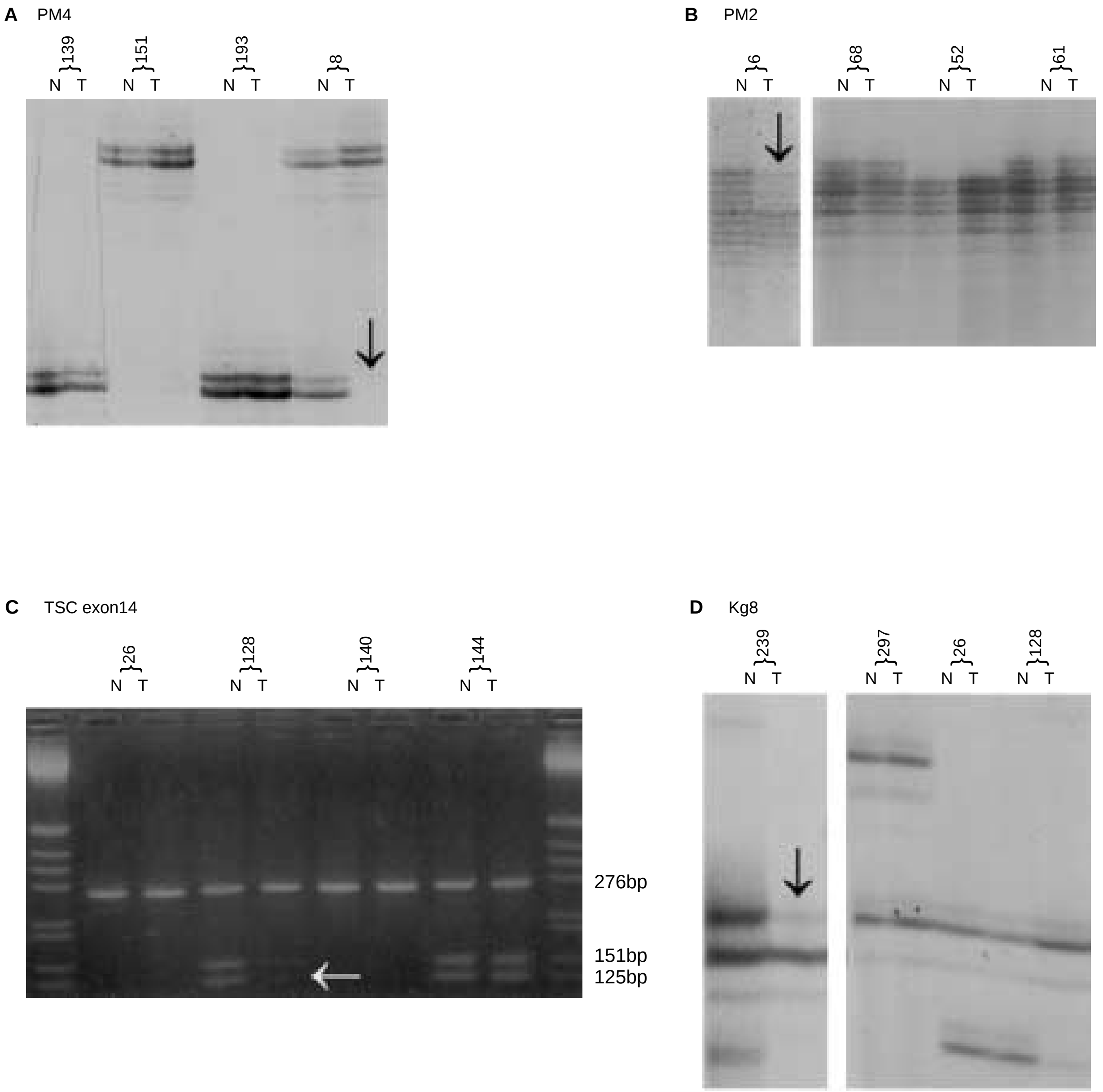

**Figure 1** Representative genotypes and examples of loss of heterozygosity. (**A**) autoradiograph showing LOH at marker PM4 (*TSC1* locus) in tumour 8. (**B**) autoradiograph showing LOH at marker PM2 (*TSC1* locus) in tumour 6. (**C**) ethidium bromide stained agarose gel showing LOH at the *TSC1* exon 14 polymorphism, E445 (1556 A >G) in tumour 128. (**D**) autoradiograph showing LOH at Kg8 at the *TSC2* locus in tumour 239

 © 2001 Cancer Research Campaign

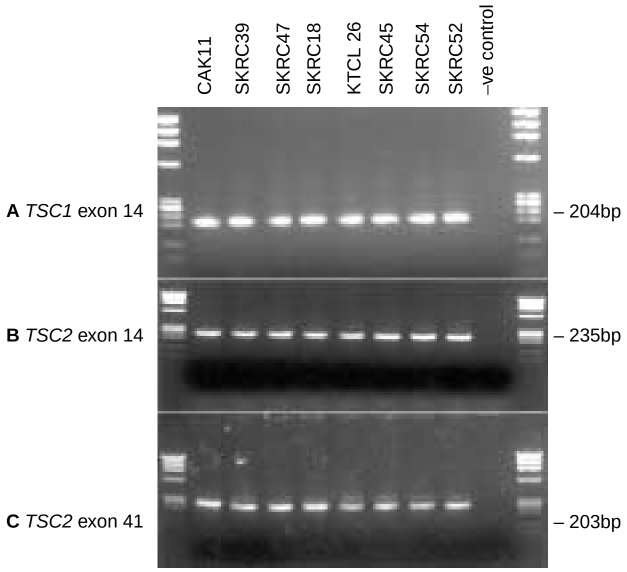

**Figure 2** RT-PCR amplification of *TSC1* and *TSC2* transcripts from 8 clear cell RCC cell lines. Expected product sizes are indicated on the right hand side. No RNA was added to the RT-PCR for the negative control lane one showed LOH (Tables 1 and 2). Of the 33 tumours informative for markers in both regions 8 showed LOH at one locus (Tables 1 and 2, Figure 1). SSCP analysis of all known coding exons (exons 3–23) and flanking intronic DNA of *TSC1* in the 7 RCCs that displayed LOH in the *TSC1* region revealed an aberrant conformer in one case, 297. However, sequencing showed this to result from a constitutional 2 bp deletion in intron 15 at bp 2218 + 71 that was considered likely to represent a non-pathogenic polymorphism. SSCP analysis in tumour 128 confirmed LOH, since one allele at a constitutionally heterozygous polymorphism in exon 14 was lost but no other mutations were detected. SSCP analysis of the coding exons 1–41 of *TSC2* in tumour 239, that showed LOH at the *TSC2* locus, did not reveal any aberrant conformers.

RT-PCR of total RNA from each of 8 independent clear-cell RCC cell lines confirmed expression of both *TSC1* and *TSC2* (Figure 2).

### DISCUSSION

Our data do not support a frequent role for *TSC1* or *TSC2* inactivation in sporadic clear-cell (with or without VHL gene inactivation) or non-clear-cell RCC. Although LOH was observed in 5 of 20 clear-cell tumours and 3 of 13 non-clear-cell tumours informative at both the *TSC1* and *TSC2* loci, apparently random allelic loss at similar frequencies has been reported in malignant tumours of the colon, breast and pancreas (Vogelstein et al, 1989; Seymour et al, 1994; Radford et al, 1995). The lack of detectable 'second hits' affecting the retained *TSC1* or *TSC2* allele makes the biological relevance of LOH difficult to assess, as this frequently involves large genomic regions that include many genes of potential importance in tumourigenesis. It remains possible that the retained *TSC1* or *TSC2* alleles in some of the RCCs studied may have been inactivated by mutations that escaped detection, such as whole exon deletions or by epigenetic mechanisms such as promoter methylation. However, expression of both *TSC1* and *TSC2* was confirmed by RT-PCR analysis in each of 8 clear-cell RCC cell lines, ruling out biallelic inactivation of either gene by such mechanisms in these cases.

Although RCC does occur in TSC it may be overdiagnosed. The histological appearances of angiomyolipoma are very variable and some lesions could be mistaken for atypical RCC (Pea et al, 1998). However, careful histopathological assessment in a number of cases showing clear cell, granular, papillary and anaplastic morphology indicate that the association between TSC and RCC is real (Robertson et al, 1996; Henske et al, 1998). Detailed immunohistochemical analysis of 6 TSC-associated RCCs has pointed to immunophenotypic differences from the majority of sporadic RCC, since 4 tumours (all showing regions of anaplastic 'spindle-cell' morphology) displayed immunoreactivity with HMB-45, a marker that also stains TSC-associated angiomyolipomatous and lymphangioleiomyomatomatous lesions, but did not show immunoreactivity for cytokeratin antibodies that are characteristically strongly positive in RCC (Robertson et al, 1996). These differences may reflect alternative molecular mechanisms in renal carcinogenesis in TSC-associated and sporadic RCC. Further molecular characterisation of TSC-associated RCC should clarify whether this is indeed the case.

### ACKNOWLEDGEMENTS

This work was supported by Cancer Research Wales, the Tuberous Sclerosis Association, Tuberous Sclerosis Alliance, Medical Research Council and the Cancer Research Campaign (CRC).

### REFERENCES

Al-Saleem T, Wessner LL, Scheitauer BW, Patterson K, Roach ES, Dreyer SJ, Fujikawa K, Bjornsson J, Bernstein J and Henske EP (1998) Malignant tumours of the kidney, brain and soft malignant tissues in children and young adults with the tuberous sclerosis complex. *Cancer* **83**: 2208–2216

Bissig H, Richter J, Desper R, Meier V, Schrami P, Schäffer, Sauter G, Mihatsch MJ and Moch H (1999) Evaluation of the clonal relationship between primary and metastatic renal cell carcinoma by comparative genomic hybridisation. *Am J Path* **155**: 267–274

Bjornsson J, Short MP, Kwiatkowski DJ and Henske EP (1996) Tuberous sclerosis-associated renal cell carcinoma: clinical, pathologic and genetic features. *Am J Path* **149**: 1201–1208

Cho M, Konishi N, Yamamoto K, Inui T, Kitahori Y, Nakagawa Y, Uemura H, Hirao Y and Hiasa Y (1998a) Genomic aberrations in renal cell carcinomas detected by restriction landmark genomic scanning. *Eur J Cancer* **13**: 2112–2118

Cho M, Konishi N, Kitahori Y, Hiasa Y, Nakagawa YI, Uemura H, Hirao Y and Oosterwijk E (1998b) Detection of DNA amplification in human renal cell carcinoma cell lines using restriction landmark genomic scanning. *Cell Mol Biol* **44**: 913–918

Clifford SC, Prowse AH, Affara NA, Buys CHCM and Maher ER (1998) Inactivation of the von Hippel-Lindau (VHL) tumour suppressor gene and allelic losses at chromosome arm 3p in primary renal cell carcinoma: Evidence for a VHL-independent pathway in clear cell renal tumourigenesis. *Genes Chromosomes Cancer* **22**: 200–209

Clifford SC, Walsh S, Hewson K, Brinke A, Green PM, Gianelli F, Pause A, Eng C and Maher ER (1999) Genomic organization and chromosomal localization of the human CUL2 gene and the role of von hippel-lindau tumor suppressor-binding protein (CUL2 and VBP1) mutation and loss in renal-cell carcinoma development. *Genes Chromosomes Cancer* **26**: 20–28

European Chromosome 16 Tuberous Sclerosis Consortium (1993) Identification and characterisation of the tuberous sclerosis gene on chromosome 16. *Cell* **75**: 1305–1315

Foster K, Prowse A, van den Berg A, Fleming S, Hulsbeek MMF, Crossey PA, Richards FM, Cairns P, Affara NA, Ferguson-Smith MA, Buys CHCM and Maher ER (1994) Somatic mutations of the von Hippel-Lindau disease tumour suppressor gene in nonfamilial clear cell renal carcinoma. *Hum Mol Gen* **3**: 2169–2173

Gnarra JR, Tory K, Weng Y, Schnidt L, Wei MH, Li H, Latif F, Liu S, Chen F and Duh FM (1994) Mutations of the VHL tumour suppressor gene in renal carcinoma. *Nature Genet* **7**: 85

*© 2001 Cancer Research Campaign* 

Henske EP, Thorner P, Patterson K, Zhuang ZP and Bernstein J (1998) Renal cell carcinoma in children with diffuse cystic hyperplasia of the kidneys. *Ped Dev Path* **2**: 270–274

Herman JG, Latif F, Weng Y, Lerman MI, Zbar B, Liu S, Samid D, Dah-Shuan RD, Gnarra JR, Marston-Linehan W and Baylin S (1994) Silencing of the *VHL* tumour-suppressor gene by DNA methylation in renal carcinoma. *Proc Natl Acad Sci USA* **91**: 9700–9704

Jones AC, Daniells CE, Snell RG, Tachataki M, Idziaszczyk SA, Krawczak M, Sampson JR and Cheadle J (1997) Molecular genetic and phenotypic analysis reveals differences between *TSC1* and *TSC2* associated familial and sporadic tuberous sclerosis. *Hum Mol Gen* **6**: 2155–2161

Kobayashi T, Hirayama Y, Kobayashi E, Kubo Y and Hino O (1995) A germline insertion in the tuberous sclerosis (*Tsc2*) gene gives rise to the Eker rat model of dominantly inherited cancer. *Nature Genet* **9**: 70–74

Kobayashi T, Urakami S, Hirayama Y, Yamamoto T, Nishizawa M, Takahara T, Kubo Y and Hino O (1997) Intragenic *Tsc2* somatic mutations as Knudson's second hit in spontaneous and chemically induced renal carcinomas in the eker rat model. *Jpn J Cancer Res* **59**: 1206–1211

Kobayashi T, Minowa O, Kuno J, Mitani H, Hino O and Noda T (1999) Renal carcinogenesis, hepatic hemangiomatosis, and embryonic lethality caused by a germline *Tsc2* mutation in mice. *Cancer Res* **59**: 1206–1211

Latif F, Tory K, Gnarra J, Yao M, Duh FM, Orcutt ML, Stackhouse T, Kuzmin I, Modi W, Geil L, Schmidt L, Zhou FW, Li H, Wei MH, Chen F, Glenn G, Choyke P, Walther MM, Weng YK, Duan DSR, Dean M, Glavac D, Richards FM, Crossey PA, Fergusonsmith MA, Lepaslier D, Chumakov I, Cohen D, Chinault AC, Maher ER, Linehan WM, Zbar B and Lerman MI (1993) Identification of the VonHippel-Lindau disease tumor-suppressor gene. *Science* **260**: 1317–1320

Onda H, Lueck A, Marks PW, Warren HB and Kwiatkowski DJ (1999) Tsc2(+/–) mice develop tumors in multiple sites that express gelsolin and are influenced by genetic background. *J Clin Invest* **104**: 687–695

Parry L, Maynard JH, Patel A, Hodges AK, von Deimling A, Sampson JR and Cheadle JP Analysis of the *TSC1* and *TSC2* tumour suppressor genes in sporadic glial and glioneuronal tumours. *Hum Genet* **107**: 350–356

Pea M, Bonetti F, Martignoni G, Henske EP, Manfrin E, Colato C and Bernstein J (1998) Apparent renal cell carcinomas in tuberous sclerosis are heterogeneous – The identification of malignant epithelioid angiomyolipoma. *Am J Surg Path* **22**: 180–187

Prowse AH, Webster AR, Richards FM, Richard S, Olschwang S, Resche F, Affara NA and Maher ER (1997) Somatic inactivation of the VHL gene in von Hippel-Lindau disease tumors. *Am J Hum Gen* **60**: 765–771

Radford DM, Fair KL, Phillips NJ, Ritter JH, Steinbrueck T, Holt MS and Doniskeller H (1995) Allelotyping of ductal carcinoma *in situ* of the breast; deletion of loci on 8p, 13q, 16q, 17p and 17q. *Cancer Res* **55**: 3399–3405

Robertson FM, Cendron M, Klauber GT and Harris BH (1996) Renal cell carcinoma in association with tuberous sclerosis in children. *J Ped Surg* **31**: 729–730

Sampson JR, Patel A and Mee AD (1995) Multifocal renal cell carcinoma in sibs from a chromosome 9 linked (TSC1) tuberous sclerosis family. *J Med Genet* **32**: 848–850

Satake H, Kobayashi T, Kobayashi E, Izumi K and Hino O (1999) Isolation and characterisation of a rat homologue of the human tuberous sclerosis gene (*Tsc1*) and analysis of its mutations in rat renal cell carcinomas. *Cancer Res* **59**: 849–855

Seymour AB, Hruban RH, Redston M, Caldas C, Powell SM, Kinzler KW, Yee CJ and Kern SE (1994) Allelotype of pancreatic adenocarcinoma. *Cancer Res* **54**: 2761–2764

Schmidt L, Duh FM, Chen F, Kishida T, Glenn G, Choyke P, Scherer SW, Zhuang ZP, Lubensky I, Dean M, Allikmets R, Chidambaram A, Bergerheim UR, Feltis JT, Casadevall C, Zamarron A, Bernues M, Richard S, Lips CJM, Walther MM, Tsui LC, Geil L, Orcutt ML, Stackhouse T, Lipan J, Slife L, Brauch H, Decker J, Niehans G, Hughson MD, Moch H, Storkel S, Lerman MI, Linehan WM and Zbar B (1997) Germline and somatic mutations in the tyrosine kinase domain of the MET proto-oncogene in papillary renal carcinomas. *Nature Genet* **16**: 68–73

Shuin T, Kondo K, Torigoe S, Kishida T, Kubota Y, Hosaka M, Nagashima Y, Kitamura H, Latif F and Zbar B (1994) Frequent somatic mutations and loss of heterozygosity of the von Hippel-Lindau tumour suppressor gene in primary human cell carcinoma. *Cancer Res* **54**: 2852–2855

Thrash-Bingham CA, Greenberg RE, Howard S, Bruzel A, Bremer M, Goll A, Salazar H, Freed JJ and Tartof KD (1995) Comprehensive allelotyping of human renal cell carcinomas using microsatellite DNA probes. *Proc Natl Acad Sci USA* **92**: 2854–2858

Urakami S, Tokuzen R, Tsuda H, Igawa M and Hino O (1997) Somatic mutation of the tuberous sclerosis (*Tsc2*) tumor suppressor gene in chemically induced rat renal carcinoma cell. *J Urol* **158**: 275–278

van Slegtenhorst M, deHoogt R, Hermans C, Nellist M, Janssen B, Verhoef S, Lindhout D, van den Ouweland A, Halley D, Young J, Burley M, Jeremiah S, Woodward K, Nahmias J, Fox M, Ekong R, Osborne J, Wolfe J, Povey S, Snell RG, Cheadle JP, Jones AC, Tachataki M, Ravine D, Sampson JR, Reeve MP, Richardson P, Wilmer F, Munro C, Hawkins TL, Sepp T, Ali JBM, Ward S, Green AJ, Yates JRW, Kwiatkowska J, Henske EP, Short MP, Haines JH, Jozwiak S and Kwiatkowski DJ (1997) Identification of the tuberous sclerosis gene *TSC1* on chromosome 9q34. *Science* **277**: 805–808

Verdorfer I, Culig Z, Hobisch A, Bartsch G, Hittmair A, Duba HC and Erdel M (1998) Characterisation of a collecting duct carcinoma by cytogenetic analysis and comparative genomic hybridisation. *Int J Oncol* **3**: 461–464

Vogelstein B, Fearon ER, Kern SE, Hamilton SR, Preisinger AC, Nakamura Y and White R (1989) Allelotype of colorectal carcinomas. *Science* **244**: 207–211

Yeung RS, Xiao GH, Jin F, Lee WC, Testa JR and Knudson AG (1995) Allelic loss at the tuberous sclerosis 2 locus in spontaneous tumours in the Eker rat. *Mol Carcinogen* **14**: 28–36

 *© 2001 Cancer Research Campaign*
